# Marine Natural Products Acting on the Acetylcholine-Binding Protein and Nicotinic Receptors: From Computer Modeling to Binding Studies and Electrophysiology

**DOI:** 10.3390/md12041859

**Published:** 2014-03-28

**Authors:** Denis Kudryavtsev, Tatyana Makarieva, Natalia Utkina, Elena Santalova, Elena Kryukova, Christoph Methfessel, Victor Tsetlin, Valentin Stonik, Igor Kasheverov

**Affiliations:** 1 Shemyakin-Ovchinnikov Institute of Bioorganic Chemistry, Russian Academy of Sciences, Miklukho-Maklaya Street, 16/10, Moscow 117997, Russia; E-Mails: kudryavtsev@ibch.ru (D.K.); evkr@mail.ru (E.K.); vits@mx.ibch.ru (V.T.); 2 G.B. Elyakov Pacific Institute of Bioorganic Chemistry (PIBOC), Russian Academy of Sciences, Prospect 100 let Vladivostoku, 159, Vladivostok 690022, Russia; E-Mails: makarieva@piboc.dvo.ru (T.M.); utkinan@mail.ru (N.U.); santalova@piboc.dvo.ru (E.S.); stonik@piboc.dvo.ru (V.S.); 3 Department of Biochemistry I, Ruhr University, Bochum 44780, Germany; E-Mail: christoph.methfessel@rub.de

**Keywords:** marine natural compounds, acetylcholine-binding protein, nicotinic acetylcholine receptors, computer modeling, radioligand assay, electrophysiology

## Abstract

For a small library of natural products from marine sponges and ascidians, *in silico* docking to the *Lymnaea stagnalis* acetylcholine-binding protein (AChBP), a model for the ligand-binding domains of nicotinic acetylcholine receptors (nAChRs), was carried out and the possibility of complex formation was revealed. It was further experimentally confirmed via competition with radioiodinated α-bungarotoxin ([^125^I]-αBgt) for binding to AChBP of the majority of analyzed compounds. Alkaloids pibocin, varacin and makaluvamines С and G had relatively high affinities (*K_i_* 0.5–1.3 μM). With the muscle-type nAChR from *Torpedo californica* ray and human neuronal α7 nAChR, heterologously expressed in the GH_4_C_1_ cell line, no competition with [^125^I]-αBgt was detected in four compounds, while the rest showed an inhibition. Makaluvamines (*K_i_* ~ 1.5 μM) were the most active compounds, but only makaluvamine G and crambescidine 359 revealed a weak selectivity towards muscle-type nAChR. Rhizochalin, aglycone of rhizochalin, pibocin, makaluvamine G, monanchocidin, crambescidine 359 and aaptamine showed inhibitory activities in electrophysiology experiments on the mouse muscle and human α7 nAChRs, expressed in *Xenopus laevis* oocytes*.* Thus, our results confirm the utility of the modeling studies on AChBPs in a search for natural compounds with cholinergic activity and demonstrate the presence of the latter in the analyzed marine biological sources.

## 1. Introduction

Nicotinic acetylcholine receptors (nAChRs) are among the most comprehensively studied ligand-gated ion channels (see reviews [1,2,3,4]). They are widespread in fish electric organs and mammalian muscles (so-called muscle type of nAChR), central and peripheral nervous systems (neuronal nAChRs) and some non-neuronal tissues (so-called “non-neuronal” nAChRs), where they perform a multitude of functions from conducting nerve-muscle transmission to participation in the different cognitive processes and regulation of inflammatory response. Involvement of distinct nAChR subtypes in different pathologies (muscle dystrophies, Alzheimer’s and Parkinson’s diseases, schizophrenia, nicotinic addiction, chronic pain) dictates the need for potent and highly selective cholinergic ligands to the respective receptor subtypes. In addition, utilization of similar new compounds as drugs in clinics is under intensive development [5].

A huge number of compounds of different chemical nature from various taxa (bacteria, plants, mollusks, chordates) are known to interact with nAChRs. Among them, the most well-known and widely used are α-neurotoxins from snake venoms (see reviews [6,7,8]). The last three decades witnessed the discovery and intensive scientific application of numerous *Conus* mollusk venom-derived conotoxins of various classes (see reviews [9,10,11]). Other marine creatures were much less studied for presence of cholinergic compounds remaining in the shadow of the latter. Among them, spirolide and gymnodimine phytoplankton toxins [12], nereistoxin from *Lumbriconereis heteropoda* annelid [13] and two ascidian alkaloids [14] should be mentioned.

**Figure 1:**
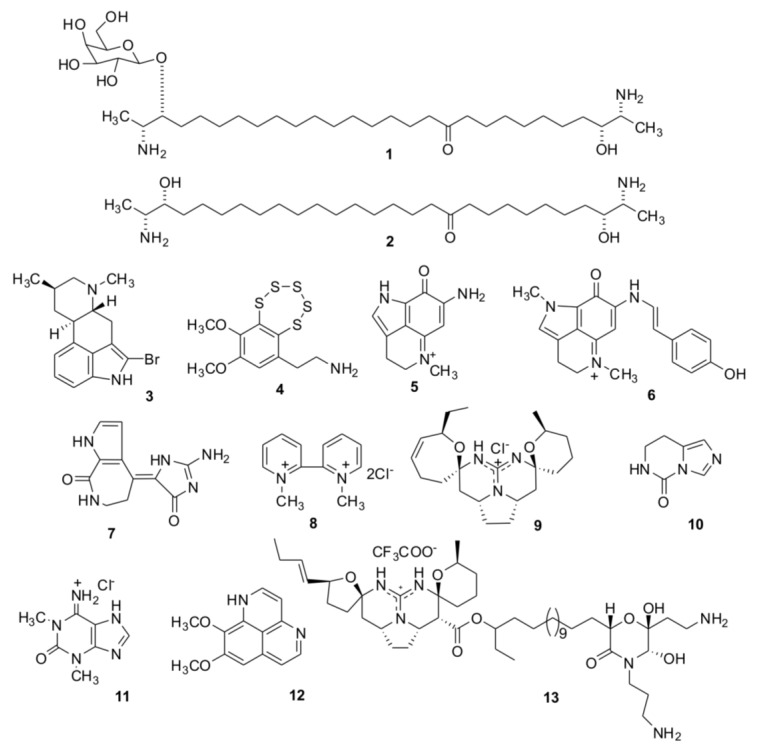
Chemical structures of compounds from marine sponges and ascidians (**1**–**13**), for which putative cholinergic activities were examined by computational and experimental methods.

Herein, we describe cholinergic properties of 13 natural low molecular weight compounds, isolated at PIBOC from marine sponges and ascidians (Figure 1). A large number of various bioactive compounds were earlier isolated from these two animal taxa, however molecular targets were not identified for the most of them. For some of them, a structural similarity to diverse cholinergic ligands (quaternary ammonium salts, heterocyclic compounds) allowed us to anticipate their possible activity towards nAChRs. To check this, we performed docking of these natural products to *Lymnaea stagnalis* acetylcholine-binding protein (AChBP) using the available X-ray structures of this protein in complexes with different cholinergic ligands. Several known AChBPs were found to be excellent structural models for the ligand-binding domains of all nAChRs (see reviews [15,16]), and now are widely used from purification of new natural cholinergic ligands [17] to design the libraries of synthetic compounds [18,19]. In the present communication, the conclusions from computer modeling were verified by efficient interaction of the studied compounds with AChBP revealed by radioligand analysis, as well as by their binding to muscle and α7 neuronal nAChRs tested by radioligand analysis and electrophysiology.

## 2. Results and Discussion

### 2.1. Isolation of Individual Compounds

Structures of the tested compounds are given on the Figure 1.

Rhizochalin (**1**), its aglycone (**2**) [20,21], pibocin (**3**) [22], and monanchocidin (**13**) [23] were for the first time isolated and described as new natural products by authors of the paper (T.M. and colleagues) from the sponge *Rhizochalina incrustata*, an ascdian *Eudistoma* sp. and the sponge *Monanchora pulchra*, respectively. Varacin (**4**) was isolated from the far-eastern ascidian *Polycitor* sp. [24]. Makaluvamines C and G (**5**, **6**) were isolated from the Australian sponge *Zyzzya fuliginosa* [25] and structurally identified with the corresponding alkaloids previously obtained from Fijian sponges belonging to the same genus [26], and an Indonesian sponge *Histodermella* sp. [27]. Debromohymenialdesine (**7**) was isolated from the sponge *Acanthella cartery* [28] and identified with the alkaloid of the sponge *Phakellia flabelata* [29]. 1,1′-Dimethyl-[2,2′]-bipyridyldiium salt (**8**) was isolated from the Far-Eastern ascidian *Botrylloides violaceus* and identified with the same compound found in the bivalve mollusk *Callista chione* earlier [30]. Crambescidin 359 (**9**) was obtained from alcoholic extract of the Australian sponge *Monanchora clathrata* and identified with the alkaloid previously isolated from the sponge *Monanchora unguiculata* [31]. 7,8-Dihydroimidazo-[1,5-*c*]-pyrimidin-5(6*H*)-one (**10**) was isolated from the Vietnamese sponge *Aplysina* sp. [32] and structurally identified with the metabolite of *Aplysina fistularis* forma *fulva* [33]. 1,3-Dimethylisoguaniniium hydrochloride (**11**) was isolated from the Far-Eastern ascidian *Syncarpa oviformis* and identified with the same compound from the sponge *Amphimedon paraviridis* [34]. Aaptamine (**12**) was obtained from extracts of the Vietnamese sponge *Aplysina* sp. [35] and identified at comparison with that from *Aaptos aaptos* [36].

### 2.2. Docking to AChBP and Analysis of Binding Parameters in Competition with [^125^I]-αBgt

We performed docking of all compounds to *Lymnaea stagnalis* AChBP using the structure of a protein with a HEPES buffer molecule in the binding site [37], the AChBP structure in complex with competitive antagonist dihydro-β-erythroidine (DHβE) [38], which is a low molecular weight alkaloid, and with the competitive antagonist α-cobratoxin [39], a snake α-neurotoxin close in structure and properties to α-bungarotoxin. In the α-cobratoxin complex, the loop C of each protomer moved away from the central axis of pentameric AChBP in comparison with its position in the HEPES bound protein. On the contrary, in the case of the complex with DHβE, its position is even more close to the central axis and is shifted down in the perpendicular direction. In our opinion, the use of three different spatial forms of AChBP for docking each of the studied compounds increases the significance of such calculations.

The results, presented in the form of calculated inhibition constants (*K_i_*), are summarized in Table 1. For almost all the compounds, the most energy-preferable position was found in the “classical” binding site corresponding to the hydrophobic pocket of nAChRs, where the cholinergic agonists and competitive antagonists are bound. However, computer docking in some cases resulted in the preferred location of the molecule in other (non-classical) binding sites. The most striking example is the 1,3-dimethylisoguaniniium hydrochloride (**11**), for which the theoretical calculated *K_i_* values for “classical” and another binding sites differed by a factor of 8 (see Table 1).

According to computational data, a high affinity to *L. stagnalis* AChBP could be expected for some compounds (**3**–**7**, **9**, **12**, **13**), and low efficiency of interaction or lack of it—for the others (compounds **1**, **2**, **8**, **10**, **11**). It should be noted that docking of almost all active compounds (**3**, **4**, **5**, **6**, **9**, **13**) to three different forms of AChBP gave similar results with predicted affinities differing by less than 5 μM. Only in case of the moderately active aaptamine (**12**) and very weak 1,1′-dimethyl-[2,2′]-bipyridyldiium salt (**8**) the predicted affinities displayed significant discrepancy, which could possibly be explained by differences in distances between the AChBP residues critical to α-cobratoxin binding, as maximal differences are observed in docking to that form of AChBP (Table 1, column C).

**Table 1:** The calculated and measured affinities of the studied compounds (see Figure 1) to *L. stagnalis* acetylcholine-binding protein (AChBP). **A**—HEPES-bound; **B**—DHβE-bound and **C**—α-cobratoxin-bound forms of AChBP respectively; *—only a part of the compound could be docked correctly because of multiple rotatable bonds; **—the binding outside “classical” agonist/competitive antagonist site is predicted; n.d.—not determined (no positive solutions). The measured affinities were obtained as follows: firstly, the respective IC_50_ values were calculated from inhibition curves (see Figure 2) using ORIGIN 7.5 and then converted into the *K_i_*s according to Cheng-Prusoff equation (see Experimental Section) with the mean ± s.e.m. values of duplicate or triplicate measurements for each compound concentration.

Compounds	Theoretical calculated K_i_ values (μM) for interaction with *L. stagnalis* AChBP			Experimental K_i_ values (μM) at interaction with *L. stagnalis* AChBP from radioligand assay
	A	B	C	
1 *	n.d.	n.d.	39	28 ± 4
2 *	n.d.	n.d.	39	130 ± 10
**3**	0.98	1.7 0.64 **	2.0	0.83 ± 0.04
**4**	1.6	0.97	0.75	0.79 ± 0.03
**5**	2.2	0.46	5.8	1.3 ± 0.1
**6**	0.05	0.04	0.97	0.55 ± 0.01
**7**	8.2	0.79 0.50 **	1.7	>1000
**8**	33	12	310	540 ± 60
**9**	0.08	0.47	0.40	27 ± 2
**10**	170	120	n.d. 320 **	>1000
**11**	150	83 11 **	350	>1000
**12**	1.5	5.3	19	3.0 ± 0.4
13 *	0.53	0.43	0.11	2.5 ± 0.4

**Figure 2:**
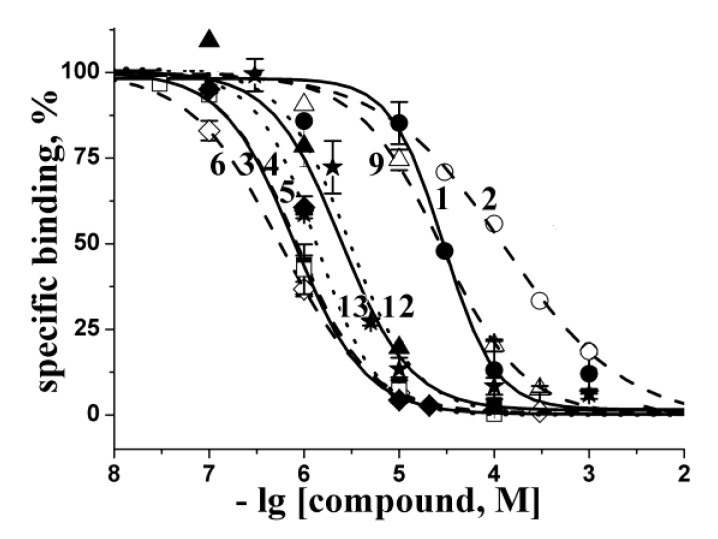
Inhibition of [^125^I]-αBgt binding to *L. stagnalis* AChBP with the most active compounds studied. Numbering the compounds corresponds to the numbering in Figure 1. The corresponding curves are marked with symbols: **1**—filled circles; **2**—open circles; **3**—filled squares; **4**—open squares; **5**—filled diamonds; **6**—open diamonds; **9**—open triangles; **12**—filled triangles; **13**—stars. Each point is a mean ± s.e.m value of two or three measurements for each concentration. The curves were calculated from the means ± s.e.m. using ORIGIN 7.5 program (see Experimental Section). The respective *K_i_* values are listed in Table 1.

The interactions, suggested by the calculations, were confirmed in radioligand assay by testing the ability of the compounds to compete with [^125^I]-αBgt for the *L. stagnalis* AChBP “classical” binding sites. The inhibition curves of the most active compounds are presented in Figure 2 and the respective K_i_ values are collected in Table 1. The compounds with the worst *in silico* docking score showed also a poor ability to compete with [^125^I]-αBgt (**1**, **2**, **8**) or were inactive with *K_i_* > 1000 μM (compounds **10**, **11**). Moreover, binding activities for some of the most active compounds (**3**–**6**, **12**, **13**) showed good agreement with the results of computer docking.

Thus, the results obtained for the majority of the studied compounds show once again sufficient validity of this computational approach for a preliminary evaluation of affinity of new ligands and even for generating assumptions about intermolecular bonds determining this affinity.

**Figure 3:**
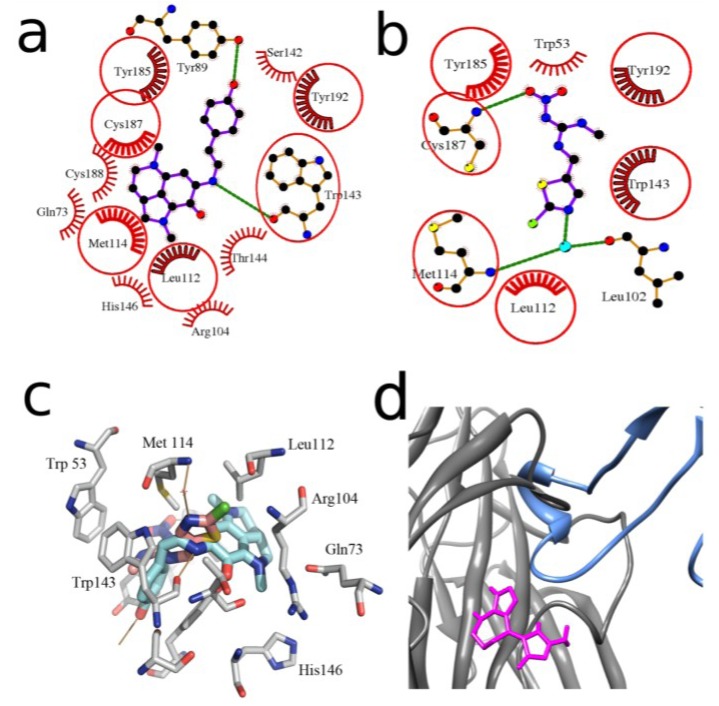
Schematic planar image of the model of makaluvamine G (**6**) docked to HEPES-bound form of *L. stagnalis* AChBP binding site (**a**) in comparison with the same presentation of the X-ray structure of the complex of the same protein with clothianidin (PDB ID—2ZJV) (**b**); intermolecular bonds in ligands are colored in magenta; the same links in the AChBP amino acid residues involved in the formation of hydrogen bonds (*green lines*) are shown in orange. The AChBP amino acid residues forming hydrophobic contacts with ligands are presented as red combs. Those of them that are the same for both ligands are encircled by *red lines*. Atoms of carbon, oxygen, nitrogen, and sulfur are colored in black, red, blue and yellow, respectively. Water molecule involved in the formation of hydrogen bonds is shown in cyan; (**c**) superposition of 3D structures of makaluvamine G (**6**), docked to AChBP and crystal structure of clothianidine AChBP complex. Carbons of makaluvamine G (**6**), clothianidine and AChBP are shown in light blue, pink and grey respectively; oxygens are shown red, nitrogens are shown blue; hydrogen bonds are brown; (**d**) superposition of α-cobratoxin structure (blue) (PDB ID—1YI5) and the best solution for debromohymenialdesine (**7**) (magenta) *in silico* docked to *L. stagnalis* AChBP (gray).

As an example, we can consider a model of the *L. stagnalis* AChBP complexed with the compound that shows the best binding parameters both in the computer docking and in radioligand assay, namely with makaluvamine G (**6**) (Figure 3a). In this schematic planar model all the ligand interactions with formation of hydrogen bonds and hydrophobic contacts with definite AChBP amino acid residues can be clearly seen. A high affinity of makaluvamine G (**6**), deduced from computer calculations, is determined by two hydrogen-bonds and 14 hydrophobic contacts with different *L. stagnalis* AChBP amino acid residues (Figure 3a).

We compared this calculated structure with the X-ray structure of the same protein complexed with clothianidin (a neonicotinoid insecticide) (Figure 3b) [40]. In that case, two hydrogen bonds (one of which with the oxygen of bound water molecule) and 10 hydrophobic interactions were revealed, 6 of which are in contacts with the same AChBP amino acid residues as in the complex with makaluvamine G. The similarity of the location of these two molecules in the binding site is obvious. Note that the affinity of clothianidin to *L. stagnalis* AChBP (*K_d_* 7.3 μM evaluated by quenching of intrinsic tryptophan fluorescence) [40] was lower than that of makaluvamine G (6) (*K_i_* = 0.55 ± 0.01 μM), perhaps because makaluvamine G fits binding site more tightly, forming more of hydrophobic contacts (Figure 3c).

Nevertheless, in two cases (compounds **7**, **9**) an essential discrepancy between the calculations and experimental data was found (see Table 1). Of course, using computer methods of calculation, one should never forget about false-positive solutions. However, another explanation of the disagreement between the calculated and measured affinities might be the interactions of tested compounds outside the “classical” agonist/competitive antagonist binding site. Such a possibility was simulated for the compound (**7**) (see Table 1). By comparing the *in silico* docked debromohymenialdesine (**7**) and the structure of three-finger toxin α-cobratoxin in their complexes with AChBP (Figure 3d) we could expect poor competition of this compound with α-bungarotoxin (very similar to α-cobratoxin), because of partially overlapping their binding sites on this target. In fact, [^125^I]-αBgt binding to *L. stagnalis* AChBP could be observed in radioligand assay even in the presence of debromohymenialdesine (**7**) as predicted by docking simulations. Analogously, an interaction of compound (**9**) outside the “classical” binding site could be supposed because of its functional activity towards α7 nAChR subtype (see Section 2.4.) which is the most close in pharmacological profile to *L. stagnalis* AChBP.

### 2.3. Analysis of Competition with [^125^I]-αBgt for Interactions with the Torpedo Californica and α7 nAChRs

**Figure 4:**
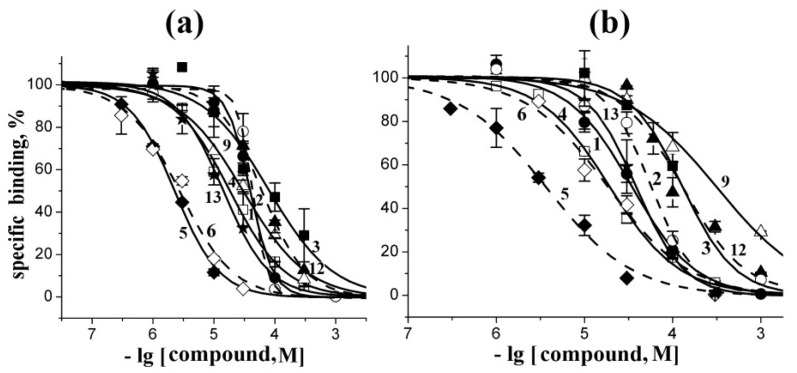
Inhibition of initial rate for [^125^I]-αBgt binding to *T. californica* nAChR (**a**) or human α7 nAChR (**b**) with the most active compounds studied. Numbering the compounds and their symbols correspond to the numbering in Figure 1 and symbols in Figure 2, respectively. Each point is a mean ± s.e.m value of two or three measurements for each concentration. The curves were calculated from the means ± s.e.m. using the ORIGIN 7.5 program (see Experimental Section). The respective *K_i_* values are listed in Table 2.

The same radioligand assay, based on competition with [^125^I]-αBgt, was used to evaluate the affinity of tested compounds towards muscle-type nAChR from *T. californica* ray and to human α7 nAChR heterologously expressed in the GH_4_C_1_ cell line. Inhibition curves for the most potent compounds and their *K_i_* values are presented in Figure 4 and Table 2, respectively.

**Table 2:** Affinity of all the studied compounds (see Figure 1) tested in competition with [^125^I]-αBgt for binding to muscle-type nAChR from *T. californica* and human neuronal α7 nAChR (see respective inhibition curves in Figure 4). The IC_50_ values were calculated using ORIGIN 7.5 and then converted into the *K_i_*s according to Cheng-Prusoff equation (see Experimental Section) with the mean ± s.e.m. values of duplicate or triplicate measurements for each compound concentration.

Compounds	*K_i_* Values (μM) on…	
	*T. californica* nAChR	human α7 nAChR
**1**	22 ± 2	33 ± 2
**2**	24 ± 2	56 ± 4
**3**	44 ± 7	130 ± 8
**4**	10 ± 1	19 ± 1
**5**	1.30 ± 0.05	3.6 ± 0.3
**6**	1.60 ± 0.17	18 ± 2
**7**	>1000	>1000
**8**	>1000	>1000
**9**	17 ± 1	310 ± 28
**10**	>1000	>1000
**11**	>1000	>1000
**12**	34 ± 1	120 ± 15
**13**	8.0 ± 1.0	38 ± 2

The compounds **7**, **8**, **10**, and **11** did not show any inhibitory activity (*K_i_* ≥ 1000 μM). A moderate potency (*K_i_* about 20–60 μM) was observed in the case of rhizochalin (**1**) and its aglycone (**2**). Somewhat less active towards both *T. californica* and human α7 nAChRs were pibocin (**3**) and aaptamine (**12**). Crambescidin 359 (**9**) has shown a twenty-fold higher activity on muscle-type receptor; in other words, it has some subtype specificity as revealed in competition with [^125^I]-αBgt. Makaluvamine G (**6**) has shown a similar ten-fold preference towards *T. californica* nAChR. The most potent competitor of [^125^I]-αBgt for the “classical” binding site on both tested receptors was makaluvamine C (**5**): it showed *K_i_* = 1.30 ± 0.05 μM on muscle-type receptor and 3.6 ± 0.3 μM on human α7 nAChR.

Summarizing these results on the activity of the studied compounds from sponges and ascidians, we ascertain that there are substances with sound cholinergic properties in these marine sources acting on both muscle-type and α7 neuronal nAChRs. Their mode of action and affinities are close to those for a number of well-known cholinergic ligands from alkaloids (nicotine, lobeline, epibatidine, d-tubocurarine, DHβE) [41], to different α-conotoxins [42,43,44], some snake venoms components (waglerins, weak toxins) [45,46] and native modulators [47] showing similar micromolar potency to the muscle and α7 nAChR subtypes evaluated in competition with [^125^I]-αBgt.

### 2.4. Electrophysiological Analysis of Effects on Functional Activity of Nicotinic Receptors

**Figure 5:**
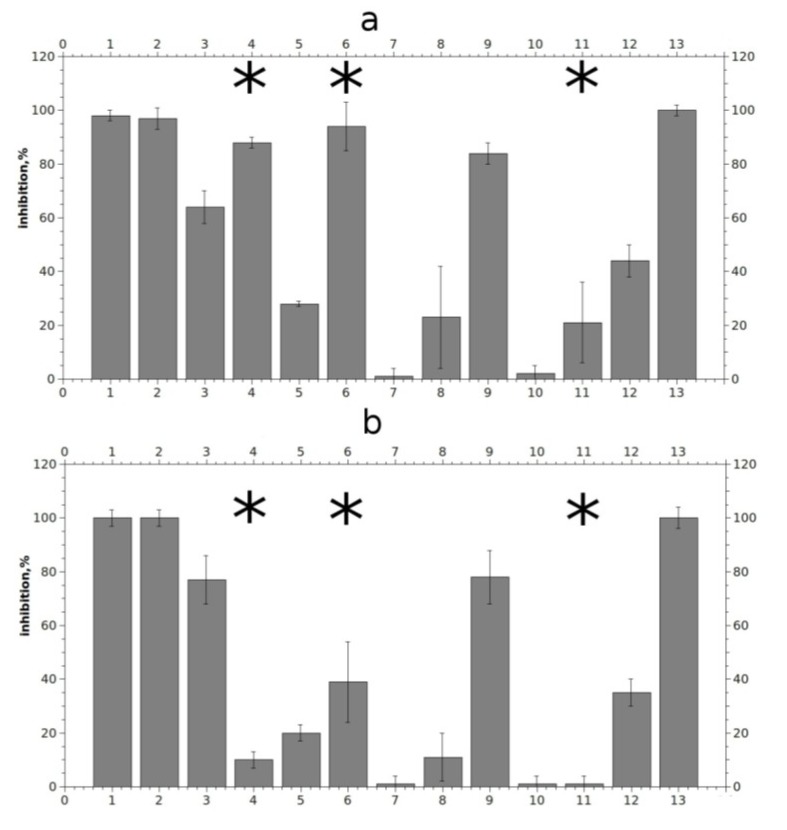
Relative inhibition of agonist-evoked current by 10 μM compounds on murine muscle-type nAChR (**a**) and on human α7 nAChR (**b**). Numbering the compounds in x axis corresponds to the numbering in Figure 1. Asterisks indicate significant (*p* < 0.05, according to Student’s test) differences between inhibition effects on murine muscle- and human α7 nAChRs for compounds (**4**), (**6**) and (**11**).

To compare the affinity found in binding studies with possible functional activity, electrophysiological measurements were performed. We applied 10 μM solutions of tested compounds to murine muscle nAChR and to human α7 nAChR expressed in *Xenopus laevis* oocytes. Figure 5 represents the inhibition activities of tested compounds in percents of maximal inhibition achieved by 15 min application of 1 μM α-bungarotoxin.

**Figure 6:**
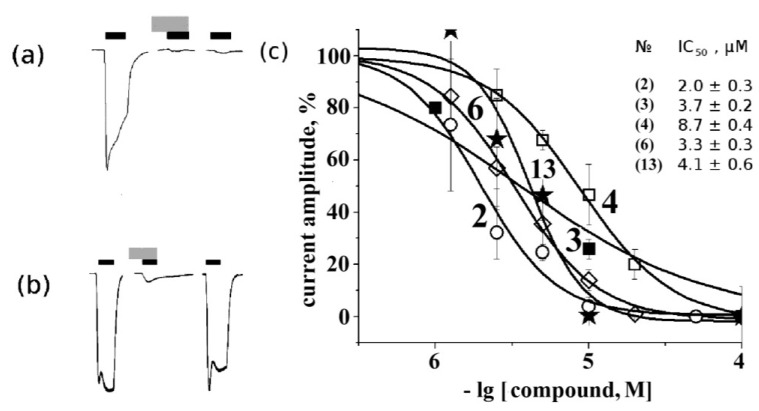
Electrophysiological measurements of 10 μM rhizochalin (**1**) (**a**) and makaluvamine G (**6**) (**b**) activity on muscle nAChR. Black and grey rectangles represent application of acetylcholine and tested compound, respectively. From left to right: control acetylcholine-evoked current, acetylcholine-evoked current in the presence of tested compound and acetylcholine-evoked current after 15 min of wash out. Current inhibition dose-response curves (**c**): concentrations of tested compounds from 1 to 100 μM were used to evaluate IC_50_ (values placed on figure according to numbering from Figure 1). The symbols used for the respective compounds are the same as in Figure 2 and Figure 4.

Compounds **1**, **2**, **6**, and **13** blocked muscle nAChR at given concentration almost completely, while compounds **3**, **4**, **9**, and **12** showed moderate activity. A weaker but still reproducible effect was shown by compound **5**, whereas only extremely weak, if any, activity was observed for compounds **7**, **8**, **10** and **11**. It is noticeable that the effects of compounds with a long non-polar carbon chain (**1**, **2** and **13**) were very slowly reversible, despite their relatively good solubility in water as seen in Figure 6a for rhizochalin (**1**) (for compounds **2** and **13** data not shown). All the tested compounds, with the exception of makaluvamine G (**6**), varacin (**4**) and to a lesser extent compound (**11**), showed similar effects both on α7 and muscle nAChR at a given concentration. Although 10 μM makaluvamine G (**6**) blocked muscle nAChR almost completely, like slowly-reversible rhizochalin and its aglycone (**1**, **2**) and monanchocidin (**13**), its inhibition has a fast off-set rate and the receptor recovered in 5 min from the end of compound application (Figure 6b). Interestingly, 10 μM varacin showed a strong inhibition of muscle nAChR, but not α7 nAChR. A difference in rates of inhibition of muscle and α7 nAChRs was observed also for compounds (**6**) and (**11**). However, even statistically significant differences in rates of inhibition should not be interpreted as a sign of selectivity towards one or another receptor subtype without further and more thorough studies.

We obtained IC_50_ values on muscle nAChR for some of the most potent compounds (Figure 6c). As can be seen, aglycone of rhizochalin (**2**) and makaluvamine G (**6**) are the most potent inhibitors of mouse muscle-type nAChR. The differences in the affinity and specificity for some compounds (**1**, **2**, **3**, **5**) found in comparison of the data obtained by radioligand analysis and electrophysiology might depend on the intrinsic features of these two methods. For the former, a crucial factor is potent and virtually irreversible binding of [^125^I]-αBgt to 2 binding sites on muscle-type nAChR and to 5 sites on α7 nAChR. On the other hand, in electrophysiology experiments the choice and concentration of agonist are of importance.

Summarizing the results of electrophysiology experiments, we can conclude that a number of analyzed compounds from marine sponges and ascidians possess functional (inhibitory) activities at micromolar concentrations. The majority of active compounds are not selective in respect to the muscle or neuronal α7 nAChRs, but some of them appear to be relatively selective towards studied receptor subtypes. However, further experiments will reveal whether they might act on heteromeric neuronal nAChRs or other Cys-loop receptors.

## 3. Experimental Section

### 3.1. Isolation of Individual Compounds

Purity of all the obtained samples were confirmed by TLC, HPLC, as well as by measurements of physical constants, while their structures were established mainly using NMR and MS data at comparison with those reported in literature [20,21,22,23,24,25,26,27,28,29,30,31,32,33,34,35,36].

### 3.2. Computer Modeling

Avogadro software was used to generate energy minimized structures of ligands [48]. Molecular dockings were performed using Autodock 4.2/Adt software [49]. Keeping in mind that some recent studies [50] discussed spatial rearrangements in the Cys-loop proteins bound to diverse types of ligands, including partial and full agonists and antagonists, we decided to perform docking to those AChBP structures which were observed in complexes with HEPES, α-cobratoxin and dihydro-β-erythroidine (PDB 1UX2, 1YI5 and 4ALX, respectively), after removing these ligands. Initially, only those *in silico* structures involving the binding sites for agonists and competitive antagonists were taken into account. Search space was centered at the C-loop, x/y/z dimensions were 120/70/50 where x is collinear with symmetry axis of the receptor and y is tangential to C-loop. The following Autodock Lamarckian genetic algorithm parameters were set: 200 dockings, evaluation number—long. Results were analyzed in respect of predicted affinity and compatibility with the αBgt binding site. LigPlot+ software [51] and PyMOL™ 1.2r2 (Molecular Graphics System, DeLano Scientific LLC, San Carlos, CA, USA) were used to analyze docked structures.

For rhizochalin, aglycon of rhizochalin and monanchocidin (**1**, **2**, **13**, respectively) we decided to dock only part of the molecule to overcome Autodock’s limitations to the number of flexible bonds. For rhizochalin and its aglycon the part from the oxo-group to the closest sphingosine head was used. The pentacyclic (crambescidine-like) part of monanchocidin represented this ligand in the docking.

### 3.3. Radioligand Assay

In competition experiments with [^125^I]-αBgt, all the compounds (in concentration range of 0.03–1000 μM) were pre-incubated 2-3 h at room temperature with the *L. stagnalis* AChBP (the final concentration of 2.4 nM) in 50 μL of buffer A (phosphate-buffered saline, 0.7 mg/mL of bovine serum albumin, 0.05% Tween 20, pH 7.5) or with the GH_4_C_1_ cells (6.5 μg of total protein with final concentration of 0.4 nM of toxin-binding sites) or *Torpedo californica* electric organ membranes (final concentration 1.25 nM of toxin-binding sites) in 50 μL of buffer B (20 mM Tris-HCl buffer, 1 mg/mL of bovine serum albumin, pH 8.0). After that [^125^I]-αBgt was added to *L. stagnalis* AChBP, GH_4_C_1_ cells or membranes to final concentration 0.1–0.2 nM and the mixtures were additionally incubated for 5 min. Binding was stopped by rapid filtration on double DE-81 filters (Whatman, Maidstone, UK) pre-soaked in buffer A (for AChBP) or on GF/C filters (Whatman) pre-soaked in 0.25% polyethylenimine (for GH_4_C_1_ cells or membranes), unbound radioactivity being removed from the filters by washout (3 × 3 mL) with the buffers A and B, respectively. Non-specific binding was determined in all cases using 2–3 h pre-incubation with 10 μM α-cobratoxin.

The binding results were analyzed using ORIGIN 7.5 (OriginLab Corporation, Northampton, MA, USA) fitting to a one-site dose-response curve by Equation:

% response = 100/{1 + ([toxin]/IC_50_)*^n^*} (1)

where IC_50_ is the concentration at which 50% of the binding sites are inhibited and *n* is the Hill coefficient. The Ki values were calculated from the experimentally measured IC_50_s by the method of Cheng & Prusoff according to Equation:

*K_i_* = IC_50_/(1 + *L*/*K_d_*) (2)

where *L* is concentration of free radioligand and *K_d_* is dissociation constant for [^125^I]-αBgt to the respective target measured under similar assay conditions. Under radioligand assay conditions used the measured *K_d_* values were 0.032 ± 0.003 nM for *T. californica* nAChR, 0.88 ± 0.19 nM for human α7 nAChR and 3.5 ± 0.6 nM for *L. stagnalis* AChBP.

### 3.4. Electrophysiology Measurements

Oocytes were obtained from benzocaine anesthetized *Xenopus* by surgically removing a part of the ovarium. Single oocytes were isolated and transferred to ND96 electrophysiology buffer. To achieve nAChR expression, each oocyte was injected with 1–5 ng of plasmid DNA containing the respective subunit genes (CHRNA1, CHRNB1, CHRND, CHRNE and CHRNA7) under control of CMV-promoter. Current recordings were performed after 36–48 h of incubation at 18 °C in ND96 solution.

All recordings were performed using the Turbo TEC-03X amplifier (npi electronic GmbH, Tamm, Germany). 3M KCl-filled recording electrodes with a resistance of about 0.1–0.5 MΩ were used. To achieve a minimum of compound consumption we used a hand-made recording chamber with approximately 25 μL volume. Membrane potential was clamped at 50–70 mV. During the experiment each oocyte was perfused with ND96 solution until the resting current reached a steady state. Then short pulses (approximately 10 s) of agonist (50 μM nicotine in the case of α7 or 10 μM acetylcholine in the case of muscle nAChR) were applied to the oocyte and then washed out with control buffer until the current returned to baseline. If the amplitude of agonist-evoked currents did not change after 5 min wash, oocytes were considered suitable for further experiments. Applications of agonist were performed every five minutes. Amplitudes of current responses to agonist in the presence of tested marine compounds were compared to the preceding agonist-induced current response. Each compound was tested on three different oocytes expressing each type of receptor (muscle or α7). In preliminary experiments the marine compounds were tested at a concentration of 10 μM, and QtiPlot software was used to display the results. For the most active compounds further measurements with concentrations of tested ligand in the range 1.25–20 μM were done, and IC_50_ values were calculated using Origin 7.5 software (OriginLab, Northampton, MA, USA).

## 4. Conclusions

In this paper, we described the interaction between nicotinic acetylcholine receptors and several low molecular weight naturally-occurring compounds from marine sponges and ascidians. Molecular docking, radioligand binding and functional electrophysiological tests were done to determine potency of these products. Makaluvamines C (**5**) and G (**6**) were the most active in radioligand binding assay, and makaluvamine G (**6**) as well as crambescidine 359 (**9**) revealed most evident selectivity towards muscle-type nAChR. In electrophysiological tests rhizochalin (**1**), aglycone of rhizochalin (**2**), pibocin (**3**), varacin (**4**), makaluvamine G (**6**), crambescidine 359 (**9**) and monanchocidin (**13**) demonstrated highest potency (in the low micromolar range) on mouse muscle nAChR. The same compounds, with the exception of (**4**) and (**6**), showed the similar affinities on human α7 nAChR. Our results indicate that some of the earlier described pleiotropic activities of the analyzed marine products may be associated with their action on nicotinic acetylcholine receptors. On the other hand, the activities revealed here by radioligand analysis and electrophysiology, as well as computer modeling in comparison with the crystalline structures of complexes of nicotinic ligands, may be useful for designing drugs against diseases associated with malfunctioning of the cholinergic system (myasthenia gravis, psychiatric and neurodegenerative diseases).
